# The Work of the Holy Ghost

**Published:** 1887-05-07

**Authors:** 


### WORDS OF CONSOLATION.

[Communications for this column should be addressed, "Chaplain," care of the Editor of THE HOSPITAL, who will gratefully welcome any suggestions or inquiries from matrons, nurses, and the staff generally.]

### XXXII.—THE WORK OF THE HOLY GHOST.

"EVERY good gift and every perfect gift is from above, and cometh down from the Father of lights, with whom is no variableness, neither shadow of turning" (James i, 17). This is true of all that we receive from God. The reason for God's gifts being continued to us is, that He changes not. Now, of course, when we first think of God's gifts, we most of us include such things as food and clothing and health, and much more that is given for our temporal welfare in answer to the prayer, "Give us our daily bread." But there is one gift, the most perfect of all, the most important of all, which we, many of us, do not include in our first thoughts of God's goodness—and that is the gift of the Holy Spirit. The Church teaches us very clearly in the Epistle and Gospel for the fourth Sunday after Easter, the connection between the best of gifts and the Holy Spirit. The Epistle commences with the passage quoted above from St. James. The Gospel (John xxi, 5) announces the conditions under which the gift of the Comforter would be bestowed upon the Church, and then what would be the results of His work in the world.

Let the sick whom we are privileged to address through this column, and those who try to comfort them, consider for awhile the Person, and the special work assigned to God the Holy Ghost. We would gladly lead some of our readers to recognise His loving hand more clearly in His dealings with their own souls. He is ever speaking to us; but in sickness His pleading is heard more distinctly than when we were deafened by the busy world, or disinclined to listen because we were too fond of sin.

The offices of the Holy Ghost are set forth in much detail in our Lord's discourse after the institution of the Sacrament of His body and blood (see John xiv, xv, xvi). These chapters should be read attentively by any who would follow us for the next few weeks. There are three points upon which we shall enlarge—1, the convictions given by the Holy Spirit; 2, His office as guide of the conscience; 3, His office as the Comforter. He is said to reprove, it should be *convince,* the world of sin, and of righteousness, and of judgment. This must be His first perceptible movement in all whom He urges to repentance; and this must take place, or otherwise He can never either guide or comfort as He desires.

Now, to convince a person is to cause him to believe something in his very heart. You may hear or read a good deal about God and about religion without being convinced. You may read or listen to these words about the Holy Ghost; but it remains to be seen whether you are convinced, satisfied, certain in your own mind that He is no less than God within you, speaking to you great truths which most deeply concern the salvation of your soul. You may be disposed to read about the Holy Ghost, or to listen willingly; and yet you may not realise His intense desire to convince you of many things, most especially of sin, righteousness, judgment. Ask Him, then, to make Himself plainer to you. Ask Him to show you why it is that the gift of His presence is so much to be desired.

(*To be continued.*)

